# High-Yield Production of Few-Layer Graphene via New-fashioned Strategy Combining Resonance Ball Milling and Hydrothermal Exfoliation

**DOI:** 10.3390/nano10040667

**Published:** 2020-04-02

**Authors:** Qingfeng Yang, Ming Zhou, Mingyang Yang, Zhixun Zhang, Jianwen Yu, Yibo Zhang, Wenjun Cheng, Xuyin Li

**Affiliations:** 1 State Key Laboratory of Tribology, School of Mechanical Engineering, Tsinghua University, Beijing 100086, China; yqf15@mails.tsinghua.edu.cn (Q.Y.); xyq142179@163.com (M.Y.);; 2 Key Laboratory for Advanced Materials Processing Technology, Ministry of Education, Beijing 100086, China

**Keywords:** resonance ball milling, hydrothermal process, few-layer graphene nanosheets

## Abstract

Graphene shows great potential applications in functional coating, electrodes, and ultrasensitive sensors, but high-yield and scalable preparation of few-layer graphene (FLG) by mechanical exfoliation method is still a formidable challenge. In this work, a novel two-step method for high-yield preparation of FLG is developed by combining resonance ball milling and hydrothermal treatment. During the resonance ball milling process, the utilization of magnetic Fe_3_O_4_ nanoparticles as a new “particle wedge” is beneficial to facilitate fragment and delamination of graphitic layers. In addition, further hydrothermal treatment can enhance ball milling product (BMP) exfoliation because of the shear force driven by the Brownian motion of various molecules at high temperature and high pressure. As expected, the two-step method can have high exfoliation efficiency up to 92% (≤10 layers). Moreover, the FLG nanosheet ink can easily achieve the formation of FLG coatings on the surface of various substrates, resulting in good electrical conductivity, which possesses potential applications in various fields including functional coating, energy storages, and electrochemical sensors, etc. Our work provides a new-fashioned strategy for mechanical large-scale production of graphene.

## 1. Introduction

Graphene, a kind of two-dimensional nanomaterial with a single atomic monolayer of sp2-bonded hexagonal carbon, is a rapidly rising star of nanomaterial science since being discovered by Novoselov K. S., Geim A. K., and Morozov S. V. in 2004 [1]. Graphene has attracted considerable attention because of its fabulous mechanical, thermal, and electrical properties, and its promising application [2]. During these years, various methods have been used to prepare graphene, which can be approximately classified into bottom-up synthesis methods including chemical vapor deposition (CVD) [3,4], solution (solvent) method [5,6], and molecular beam epitaxy (MBE) [7,8], and top-down methods such as mechanical exfoliation (including the micromechanical cleavage method [1], ultrasonic method [9,10,11], and ball milling method [12], etc.) [1,13,14,15,16], electrochemical method [17,18], oxidation-reduction method [19,20], and intercalation exfoliation method [21,22], etc. The top-down methods are more likely to achieve scalable production compared with bottom-up methods [23]. Aside from quality, the exfoliation efficiency of graphene is another important factor limiting its development and application. Considering good quality and high yield, ball milling is an ideal approach to prepare graphene [24,25,26]. The mechanism of graphene exfoliation by ball milling has revealed that the utilization of an auxiliary reagent can facilitate delamination of graphite into graphene nanosheets [24]. Thus, in order to improve the exfoliation efficiency, various auxiliary reagents were used to assist ball milling, such as dry ice [27,28], oxalic acid [25], melamine [29,30], cellulose [31], sulfur [32], and aryl diazonium salt [33], etc. Although researchers have made great progress in this field, the ball milling method still faces some problems, such as low production and long production time [34,35].

Hydrothermal treatment is a thermo-chemical conversion technique resulting in efficient hydrolysis and pyrolysis with high temperature and high pressure [36,37]. During the hydrothermal process, the original layered structural materials can be exfoliated into few-layer materials by thermo-chemical intercalation, which provides an important method for the preparation of low-dimensional materials [36,37,38,39,40]. In 2014, Liu et al. proposed that single- or few-layer graphene is produced by hydrothermal exfoliation of graphite [41]. Mojtaba Ahmadi et al. reported on the hydrothermal-assisted ball milling approach for scalable production of high-quality functionalized MoS_2_ nanosheets [42]. Ping Liu et al. presented an energy-saving and metal-free method to obtain well crystalized multilayer graphene (2–10 layers) from activated carbon via an ammonia-assisted hydrothermal route at 200 °C [39]. Although a few works reported the successful preparation of graphene by the hydrothermal method, it is difficult to realize batch production of graphene using only the hydrothermal method.

**Figure 1:**
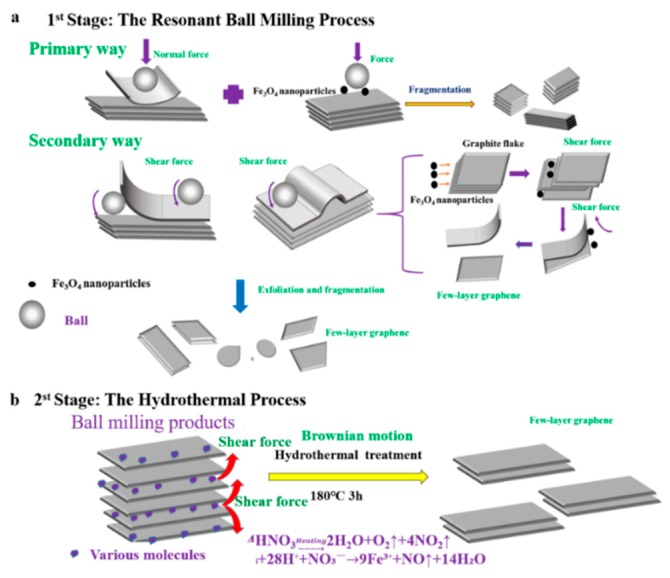
Illustration mechanism of the prepared few-layer graphene (FLG) nanosheets by two-step method with Fe_3_O_4_ nanoparticles. (**a**): the mechanism of exfoliated by the resonant ball- milling with Fe_3_O_4_ nanoparticles, (**b**): the mechanism of exfoliated by the hydrothermal treatment.

Here, in this paper, a new-fashioned two-step method combining resonance ball milling and hydrothermal treatment was developed for high-yield preparation of few-layer graphene. During the resonance ball milling process, the graphite flakes can facilitate delamination by the fragmentation and exfoliation effects due to the utilization of magnetic Fe_3_O_4_ nanoparticles as new “particle wedges”. Then, in the hydrothermal process, the ball milling products (BMPs) with a small size and a fluffy structure can be easily stripped into high quality graphene by molecule intercalation (Figure 1). The results showed that the two-step method had a high exfoliation efficiency up to 92% (≤10 layers) and a high output rate of 85.26%, which are both attributed to the strong shear forces generated from the collision of Fe_3_O_4_ particles with graphite flakes during resonance ball milling process and the Brownian motion of various molecules in the hydrothermal process. The two-step method has the advantages of high exfoliation efficiency, high output rate, and less ball milling time compared to the reported literatures (Table S2) [34,35]. This provides a new-fashioned strategy for the mechanical large-scale production of graphene. Furthermore, the few-layer graphene (FLG) nanosheet ink can easily form films that have good electrical conductivity on various substrates, which could have potential applications in various fields including functional coating, energy storages, and electrochemical sensors, etc.

## 2. Experimental Section

### 2.1. Chemicals and Materials

Expanded graphite was purchased from Qingdao Baixing Graphite Co., Ltd. (Shandong, China). Zirconia balls (4 mm and 6 mm diameter) were purchased from Shenzhen Yike Gringing Material Co., Ltd. (Guangdong, China). N-Methyl pyrrolidone (NMP) was purchased from Beijing Solarbio Science and Technology Co., Ltd. (China). Analytical reagent (AR) Nitric acid (HNO_3_) (65%~68% purity, Cat No. 7697-37-2) was purchased from Sinopharm Chemical Reagent Beijing Co., Ltd. (Beijing, China). Fe_3_O_4_ powder (particle size about 100 nm) was purchased from Qinghe County Xingye Metal Materials Co., Ltd. (Hebei, China). Yellow Smoke Remover was purchased from Suzhou Qingkong Environmental Protection Technology Co., Ltd. (Jiangsu, China).

### 2.2. Instruments

Ring spring resonance ball mill equipment was self-designed by the research group. The equipment had higher resonance vibration strength, higher output, higher milling efficiency, a simpler process, produced less noise, and made it easier to achieve scale compared to conventional ball milling equipment [43]. The vibration amplitude of the equipment was 11 mm and the vibration frequency was 16 Hz. When the filling rate in the resonant cavity was above 80%, the ball mill could reach the resonance frequency. During the resonance ball milling, the collision and vertical impacts were intense, with an excellent fragmentation and exfoliation effect. Scanning electron microscope (SEM) images were taken on a LYRA3 Focused ion-beam scanning electron microscope system (TESCAN. Q. S, Brno, Czech Republic). Transmission electron microscopy (TEM) images were taken by an H-7650 instrument (Hitachi, Japan). High-resolution transmission electron microscopy (HRTEM) images were obtained on a JEM2010 high-resolution field-emission transmission electron microscope at 200 KV (JEOL, Japan). X-ray photoelectron spectroscopy (XPS) was performed using a PHI system (Quantera II Ulvac-Phi Inc, Kanagawa, Japan). Fourier transform infrared (FTIR) spectra were recorded on a Nicolet Ncxus670 spectrophotometer (Madison, USA). X-ray powder diffraction (XRD) measurements were collected on a Bruker D8/Aduance system (Karlsruhe, Germany). Raman spectra were obtained on a LabRam-HR/VV (JY, France) at an excitation wavelength of 514 nm. Sheet resistance of the graphene nanosheet films was measured with a four-point probe resistance tester (M3, Suzhou Jingge Electronic Co., Ltd., China). The DC power (MN-3010D, Shenzhen Zhaoxin electronic instrument equipment co. Ltd., China) was used to light the LED.

### 2.3. Synthesis of Few-Layer Graphene

To prepare few-layer graphene, 20 g of expanded graphite and 2.5 g Fe_3_O_4_ nanoparticle were milled for 6 h in a ring spring resonance ball mill (the mass ratio of zirconia spheres with diameters of 6 mm and 4 mm is 1:3). Then, each 1 g of milled powder and 30 mL of concentrated HNO_3_ were transferred to a polytetrafluoroethylene (PTFE) high pressure reactor and heated at 180 °C for 3 h. Finally, the FLG nanosheets were obtained by repeated solution washing under centrifugation at 8000 rpm to remove the unreacted nitric acid. The centrifuge precipitate was freeze-dried to obtain the FLG nanosheets, and the weight ratio between the processed material and the starting expanded graphite flakes (up to 20 g) was 85.26%. In addition, the nitrogen oxides were removed by Yellow Smoke Remover (commercial product). The experimental procedures of the control groups are shown in the supporting materials (Table S1).

### 2.4. Preparation of Graphene Conductive Coatings

The FLG nanosheet ink (10 mg/mL) was prepared by dissolving the graphene nanosheets in ethanol. A series of graphene conductive coatings were prepared by brush coating, spin coating, or suction filtration of FLG nanosheet ink on a variety of substrate (e.g., copper wires and their PVC shell, plastic pipe, plant leaves, A4 paper, polyethylene glycol terephthalate(PET) film, and PTFE membrane filter) (for detailed procedures, see Supporting Information).

## 3. Results and Discussion

### 3.1. Characterizations of the FLG Nanosheets

Few-layer graphene nanosheets were investigated by different characterization methods. Apart from the verification of graphite exfoliation, these techniques help us to identify the exfoliated graphene properties.

**Figure 2:**
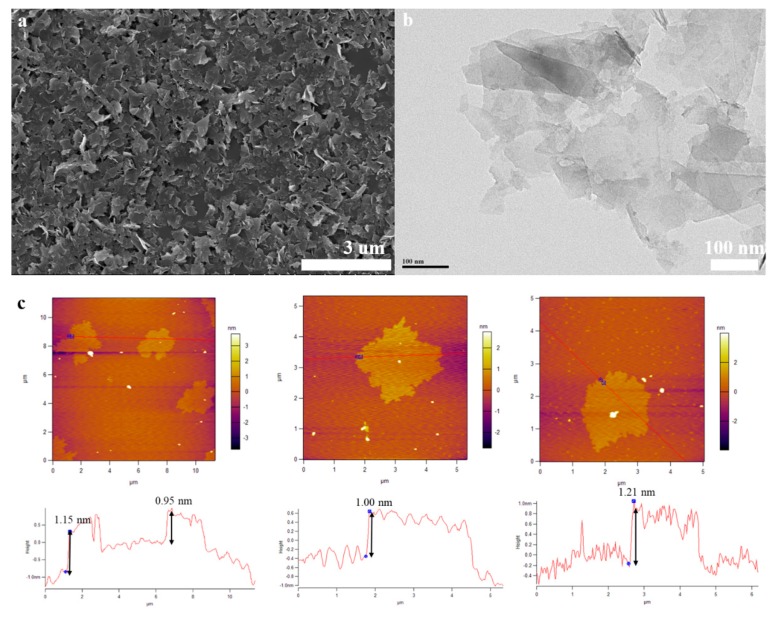
(**a**): SEM image of the FLG nanosheets, (**b**): TEM image of the FLG nanosheets, (**c**): AFM image of the FLG nanosheets.

Figure 2a and Figure S3d,e display SEM images of the FLG nanosheets. These results indicate that the FLG nanosheets have a good homogenous shapes of flakes and less wrinkle. Clear morphology of graphene sheets makes it possible to measure the size of exfoliated sheets in any directions. Therefore, the obtained lateral dimensions of 100 random nanosheets were accounted by the nanomeasure software in Figure S3e,f, and the result show that the nanosheets have a lateral average size of about 250 nm.

**Figure 3:**
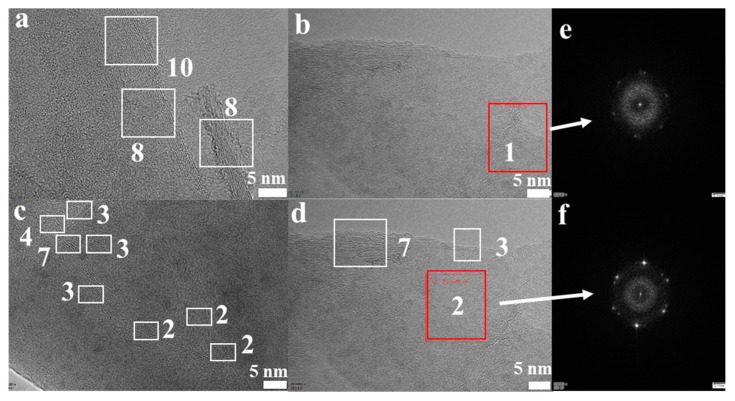
(**a**–**d**) High-resolution transmission electron microscopy (HRTEM) images of the FLG nanosheets; (**e**,**f**) the images of the measured electron diffraction pattern.

**Figure 4:**
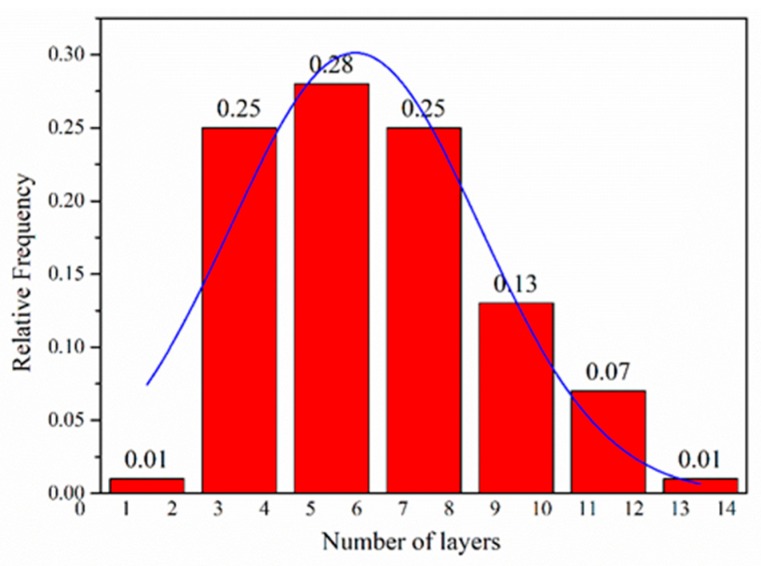
Flake thickness distribution measured using HRTEM analysis of the FLG nanosheets.

The morphological features of the FLG nanosheets were confirmed by TEM and HRTEM (Figure 2b, Figure 3 and Figure S4d). The results revealed that the majority of the nanosheets showed edge layers of less than 10 layers, and the single-layer and two-layer graphene are observed in Figure 3e,f, respectively. Well-defined edges of the FLG nanosheets make it possible to count the number of layers in the HRTEM images. Thus, thickness distribution was obtained by counting the layers at the edges of the 100 edges of the FLG nanosheets. The result showed that the graphene with less than ten-layers made up to 92% of the exfoliated FLG nanosheets, as showed in Figure 4. This result is consistent with the Raman and AFM tests (Table S3) [44]. It is worth mentioning that the exfoliation efficiency calculation is based on no separation treatment products, both in HRTEM and AFM.

The morphological properties of the FLG nanosheets were also identified by AFM (Figure 2c and Figure S5d). The thicknesses of about 1.15 nm, 0.85 nm, 1.00 nm, and 1.21 nm has been measured for these selected sheets in Figure 2c. Considering the theoretical thickness of 0.34 nm for each graphene layer, the number of layers per flake can be calculated [45]. While, due to various reasons like the folding and wrinkles of the graphene as well as instrument offset, the step heights of monolayer graphene are usually considered to be less than 1 nm in AFM studies. Thus, for more accuracy, we assumed that the flakes with heights shorter than 1 nm are less than three-layer graphene [46]. Thus, the results indicated that these were related to monolayer and multilayer graphene in AFM images, Figure 2c and Figure S5d. To better describe the two-step method exfoliation efficiency, the statistical thickness analysis of more than 100 graphene sheets were measured in AFM images. It is observed that the percentage of FLG nanosheets with thicknesses less than 3.5 nm are 92% in Figure S6. Thus, the result indicates that the majority of exfoliated FLG nanosheets were less than ten-layer graphene. Considering the previous assumption, it can be concluded that the FLG nanosheets were successfully separated from the graphite. The AFM result is consistent with HRTEM and Raman tests.

**Figure 5:**
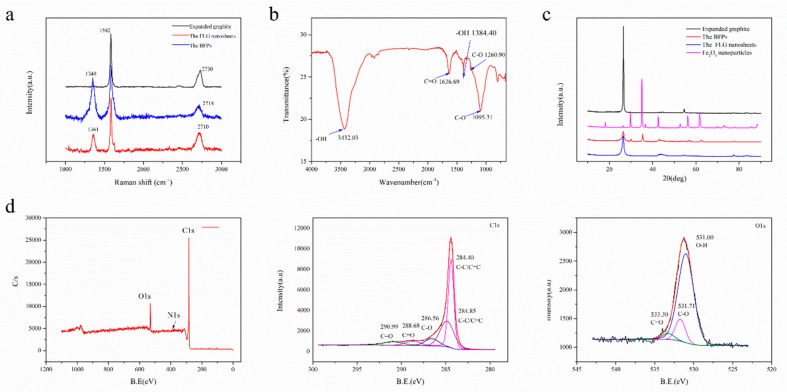
(**a**) Raman characterization of the expanded graphite, the products were only obtained by ball milling with Fe_3_O_4_ nanoparticles (BFPs) and the FLG nanosheets, (**b**) FTIR spectra of the FLG nanosheets, (**c**) XRD patterns of the expanded graphite, the Fe_3_O_4_ nanoparticles, the BFPs, and the FLG nanosheets, (**d**) X-ray photoelectron spectroscopy (XPS) spectra of the FLG nanosheets.

Raman spectroscopy was also used to check the structural characteristics of the FLG nanosheets (Figure 5a). It can reveal the relationship between the intensities of the D (I_D_), G (I_G_), and 2D (I_2D_) bands present in graphene [47]. The ratio of I_D_/I_G_ is 0.30 and the ratio of I_2D_/I_G_ is 0.35 in the FLG nanosheets. The activation of the weak D peak in the FLG nanosheets is due to the increased defects and functional groups [48]. The results suggest that the obtained nanosheets were composed of few-layer graphene. It is well known that the shapes of the 2D Raman bands (around 2700 cm^−1^) reflect the thickness of graphene [44]. The numbers of layers (N_G_) in the FLG nanosheets were calculated according to the equation described by Coleman et al. [44,49], using the information obtained from the 2D band positions. At least 20 individual Raman spectra of FLG nanosheets were used to analyze the calculations. The results showed that the average thickness of the FLG nanosheets was 7–8 layers, and the error is approximately ±1.5 layers (for detail information, see Supporting Information) [44].

Further, FTIR and XPS were performed in order to characterize the analysis of the functional groups in the obtained products. Figure 5b shows the FTIR spectrum of the FLG nanosheets. The results indicate that there were some hydrophilic groups on the surface of the FLG nanosheets due to the oxidation of nitric acid in the hydrothermal process. According to Figure 5b, the peak at ~3432 cm^−1^ is related to the hydroxyl group, which is attributed to the stretching vibrations of the O-H bond (peaks between 3300 and 3600 cm^−1^). The peaks at ~1095 cm^−1^ and 1260.90 cm^−1^ are because of the stretching vibrations of the C-O bond. The peak at ~1636.99 cm^−1^ is attributed to the stretching vibrations of the C=O bond [50,51].

Figure 5d shows the qualitative content and type of oxygen groups in the FLG nanosheets (Table 1). There were five main components with different binding energies in the C1s core-level spectra that were fitted with Gaussian–Lorentzian peaks: 284.40 eV (carbon bonds, sp^2^), 284.85 eV (carbon bonds, sp^2^), 286.56 eV (C-O bond), 288.68 eV (C=O bonds), and 290.99 eV (C=O bonds) [25,50,52]. There were three main components with different binding energies in the O1s core-level spectra that were fitted with Gaussian–Lorentzian peaks: 531.00 eV (O-H bonds), 531.713 eV (C-O bonds), and 533.30 eV (C=O bonds) [11,16,25,50,52]. Furthermore, the C/O ratio of the FLG is consistent with the elementary analyzer, which is 8.05~9.90 (Table 2). Corresponding to the above FTIR test results, it is proven that the surface of the FLG nanosheets contains some hydrophilic groups, which is good for the dispersion of FLG nanosheets in pure water (Figure S7). As showed in Figure S7, the FLG nanosheet dispersion in pure water is highly stable for over 30 days.

In a word, few-layer graphene with high quality was successfully produced from expanded graphite at high yield using the new-fashioned two-step method with Fe_3_O_4_ nanoparticles, which has some hydrophilic groups on the surface.

### 3.2. Validation Confirmation of the Two-Step Method with Fe_3_O_4_ Nanoparticles

**Figure 6:**
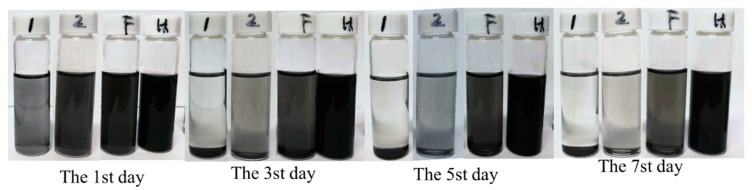
Image of the prepared powders in N-Methyl pyrrolidone (NMP) with the concentration of 0.1 mg/mL: the products were only obtained by ball milling without Fe_3_O_4_ nanoparticles (OBPs, Marked by the number 1), the products were obtained by the two-step method without Fe_3_O_4_ nanoparticles (BHPs, Marked by the number 2), the BFPs (marked by the letter F) and the FLG (marked by the letter H).

**Figure 7:**
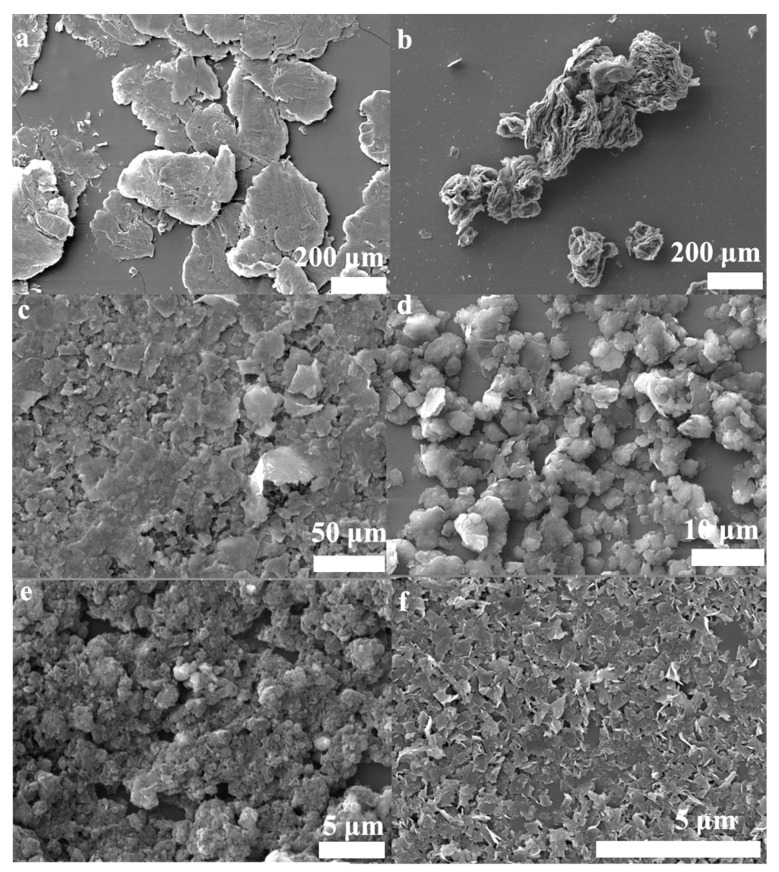
SEM images of the powders; (**a**) Expanded graphite, (**b**) the products were only obtained by hydrothermal treatment( OHPs), (**c**) the OBPs, (**d**) the BHPs, (**e**) the BFPs, (**f**) the FLG nanosheets.

In order to directly compare the stripping efficiency of these methods, the powders were dispersed in NMP solvent with a concentration of 0.1 mg/mL and were prepared at different experiment conditions (Table S1). The results showed that the samples’ sedimentation velocities were gradually decreased from left to right, as showed in Figure 6. The samples’ sedimentation velocities are related to flakes size, shape, and thicknesses, ect. [53]. The slower sedimentation velocity may reveal that there is more few-layer graphene in the solution, which can be testified from the characterization (SEM, Figure 7; TEM, Figure S4; and AFM, Figure S5). Meanwhile, it was found that the color of the solution in tube H remained black after 30 days (Figure S3g). As can be seen from Figure 7 and Figure S3, the lateral size of the graphene sheets has reduced gradually from Figure 7a–f and Figure S3a–d. These results indicate that the two-step method with Fe_3_O_4_ nanoparticles has an excellent crushing effect. Hydrothermal assisted resonant ball milling mechanisms could be considered to explain the formation of the smallest lateral size in the FLG nanosheets. The C-C bonds of the graphite flakes is intense, cracked by the motion of the grinding balls due to the addition of the Fe_3_O_4_ nanoparticles as “particle wedges” during the resonant ball milling process. Furthermore, the BMPs are stripped and broken by violent Brownian motion at high temperature and high pressure in the hydrothermal treatment stage [54]. In comparison to the products (Figure S4a–c), The TEM image of the FLG nanosheets illustrated in Figure S4d was completely transparent to the electron beam, which demonstrates the thin structure of the FLG nanosheets. Therefore, the transparency of graphene in the TEM images confirms that the FLG nanosheets were successfully separated from to graphite by the two-step method with Fe_3_O_4_ nanoparticles. 

Compared with the powders (Figure S5a–d), the step heights were gradually reduced and were located across the red line in the AFM images. The results show that the FLG nanosheets possess the thinnest step height. Apart from the AFM characterization, Figure 5a displays the Raman spectrum of the expanded graphite, the BMPs, and the FLG nanosheets. The intensity of the D peak of the FLG nanosheets is obviously weakened when free Fe_3_O_4_ nanoparticles are removed by nitric acid in the hydrothermal process. Compared to the original expanded graphite, the 2D peak has a shift from 2730 cm^−1^ of the expanded graphite spectra to 2710 cm^−1^ of the FLG nanosheets, which demonstrates the successful exfoliation from the expanded graphite into the FLG nanosheets.

Furthermore, XRD was performed to investigate the phase change of graphite flakes after exfoliation. As shown in Figure 5c, the XRD diffraction pattern of graphite exhibits a strong (002) peak at 26.5°, corresponding to an interlayer d-spacing of 0.34 nm [27]. In the case of the obtained powders by resonant ball milling with Fe_3_O_4_ nanoparticles, it has the characteristic peaks of a rather weak (002) peak and Fe_3_O_4_ nanoparticles. The intensity of the (002) peak is obviously raised, and the bands related to Fe_3_O_4_ nanoparticles almost disappear when free Fe_3_O_4_ nanoparticles were removed by nitric acid in the hydrothermal process for the FLG nanosheets. However, the height of the (002) peak of the prepared FLG nanosheets is apparently lower than the expanded graphite and its half-peak width gets broader. These results indicate that there is a high degree of exfoliation in the two-step method with Fe_3_O_4_ nanoparticles. The weakened intensity of the XRD peak through exfoliation of graphite to graphene has been reported in the literature [27,55]. Thus, according to the above different characterization results, it is proven that the two-step method with Fe_3_O_4_ nanoparticles has high stripping efficiency.

### 3.3. Mechanism of Preparation of the FLG Nanosheets

A possible synergistic mechanism of resonance ball milling and hydrothermal stripping is illustrated in Figure 1. The ball milling process using Fe_3_O_4_ nanoparticles could be explained as a multi-steps mechanism, illustrated in Figure 1a. Firstly, the graphite flakes are exfoliated by normal force that overcome the interlayer Van der Waals force between the graphite; and these flakes also are cracked into smaller ones by being hit by the motion of the grinding balls and the Fe_3_O_4_ nanoparticles. The fragmentation effect is advantageous for exfoliating the graphite due to the lower van der Waals force between the smaller-sized graphite layers relative to the large-sized graphite layers [24]. Secondly, the graphite flakes are unzipped by shear force that can promote the relative motion between two graphite layers. During the resonant ball milling process, the strong shear force is generated by intense collisions by the utilization of magnetic Fe_3_O_4_ nanoparticles as new “particle wedges”, which can facilitate delamination of graphitic layers. The Fe_3_O_4_ nanoparticles collided and moved in a random direction in the ball milling process. A considerable portion of the nanoparticles collide with the graphite flakes perpendicular to their C-axis direction. This can promote a fragmentation and exfoliation effect, resulting in more few-layer graphene nanosheets through the “particle wedge” role of the Fe_3_O_4_ nanoparticles.

Furthermore, the BMPs can be easily stripped into high quality graphene by molecule intercalation in the hydrothermal process, which will overcome the low van der Waals force between the layers for the small size and fluffy structure; illustrated in Figure 1b. At high temperatures and high pressures, nitric acid is involved in chemical reactions to produce certain amounts of gas molecules (the chemical reaction Equations (1) and (2)); and the violent Brownian motion of graphite flakes will be simultaneously caused by the violent thermal motion of various molecules that contain gas molecules, water molecule, HNO_3_ molecules, and Fe(NO_3_)_3_ molecule [54]. In the meantime, the fluffy layered structure of BMPs will fluctuate and be thermally agitated. Thus, molecules can intercalate randomly into the thermally agitated interlayers of BMPs from the lateral of products. Then, the BMPs can be easily exfoliated into FLG nanosheets by the shear forces driven by the Brownian motion of various molecules [41,54]. Therefore, the shear effect induced by the Brownian motion can also facilitate delamination of graphitic layers, just like what has been revealed in the reported literature [41]. Meanwhile, during the hydrothermal process, the Fe_3_O_4_ nanoparticles used for auxiliary grinding could be removed by nitric acid, and the surface of the FLG nanosheets could be partially oxidized by the concentrated nitric acid. Hence, the FLG nanosheets could be dispersed in pure water for hydrophilic groups, which are produced in the hydrothermal process (the characterization of XPS and FTIR). It should be pointed out that the unreacted nitric acid can be removed by washing with water or alkali neutralized, and the nitrogen oxides can be remove by Yellow Smoke Remover (commercial product).

(1) $$4{\mathrm{HNO}}_{3}\overset{\mathrm{Heating}}{\to }2H_{2}O+O_{2}↑+4{\mathrm{NO}}_{2}↑$$

(2) $$3{\mathrm{Fe}}_{3}O_{4}+28H^{+}+{\mathrm{NO}}_{3}^{-}\to 9{\mathrm{Fe}}^{3+}+\mathrm{NO}↑+14H_{2}O$$

To confirm the synergistic effect of the two-step method with Fe_3_O_4_ nanoparticles, some control experiments were carried out under the same experimental conditions (Table S1). The results show that, in the two-step method without Fe_3_O_4_ nanoparticles, a much larger lateral size and thick step height of the products (15.74~82.60 nm, Figure S5b) were obtained (step heights ≤ 6.0 nm in the exfoliation with Fe_3_O_4_ nanoparticles, Figure S6). For the exfoliation systems without ball milling, the result shows that the bulk graphite was not stripped into few-layer graphene, while the structure only became fluffy (Figure 7b). Compared with the exfoliation systems without hydrothermal treatment, the results indicate that the exfoliation efficiencies are also much lower than the two-step method with Fe_3_O_4_ nanoparticles. All the results of the above control experiments confirmed that the two-step method with Fe_3_O_4_ nanoparticles constructed a good system for expanded graphite exfoliated into few-layer graphene. Furthermore, our approach may provide a novel strategy for the scalable exfoliation of other two-dimensional nanomaterials such as MoS_2_, WS_2_, BN, ect.

### 3.4. Conductivity of the FLG Nanosheets Films

**Figure 8:**
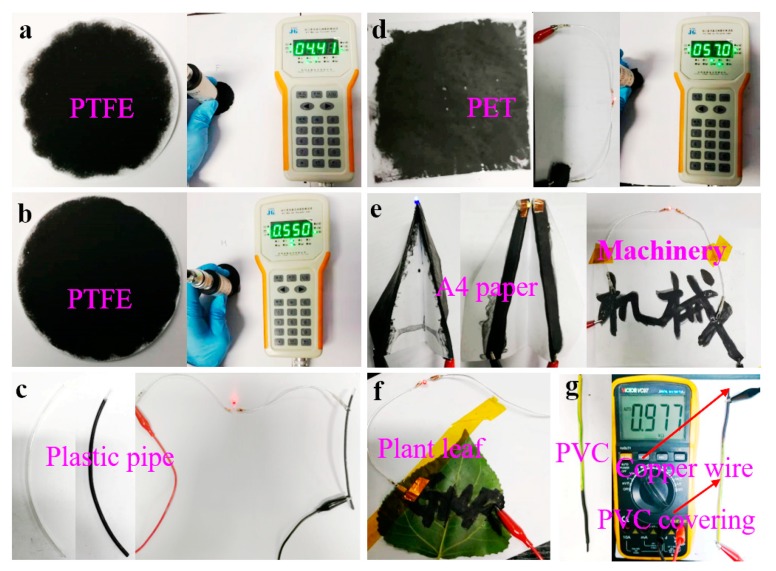
Photos of powders on various substrates: (**a**) PTFE filtered with the BFPs; (**b**) PTFE filtered with the FLG nanosheets; (**c**) conductive trace of the FLG nanosheets brushed on plastic pipe; (**d**) the FLG nanosheets filmed on PET by spin coating; (**e**,**f**) the FLG nanosheets filmed on A4 paper and plant leaves by brush coating; (**g**) conductive trace of the FLG nanosheets brushed on copper wire.

Figure 8 shows photographs of the obtained powders on various substrates. The results show that the FLG nanosheet ink had the formation of uniform films that possess good electrical conductivity (Table S4). The sheet resistances of the obtained films were tested by M3 four-point probe resistance. The result indicated that the spin coating on PET had the lowest average sheet resistance of ~51.80 Ω/sq due to its dense nanosheet film (the SEM image in Figure S8 and Table S4). Therefore, the prepared graphene ink may find potential applications in various fields including functional coating, energy storages, and electrochemical sensors, etc.

## 4. Conclusions

In this research, FLG nanosheets were prepared by a new-fashioned two-step method combining novel magnetic nanoparticle assisted resonance ball milling and hydrothermal treatment. Various characterization techniques successfully demonstrated the preparation of high quality FLG nanosheets. The strong shear forces generated by the intense collision of Fe_3_O_4_ particles with graphite flakes, and driven by the Brownian motion of various intercalated molecules, lead to enhanced exfoliation efficiency of the graphite flakes. The results showed that the exfoliation efficiency achieved can be up to 92% (≤10 layers) without purification, and high output can achieve up to 85.26% efficiency. Therefore, the method using particle wedge assisted ball milling and hydrothermal treatment could be an applicable technique for large-scale production. However, for large-scale graphene industrial production, there are two points that should be researched in further studies on this two-step method, which are the effect of wedge particles with different surface chemistry, particle size (from nano to micro scale) and morphology, and the effect of intercalation with different eco-friendly intercalation agents.

## Figures and Tables

**Table 1:** The mean atomic concentrations of FLG nanosheets were measured 3 times by XPS.

C1s	N1s	O1s
90.23	0.66	9.11

**Table 2:** The mean element content of FLG nanosheets were measured 3 times by elemental analyzer.

C1s	N1s	O1s
88.51	0.39	11.00

## Supplementary Materials

The following are available online at https://www.mdpi.com/2079-4991/10/4/667/s1, Table S1. Experimental conditions for preparation of graphene, Table S2. A comparison between different ball-milling and hydrothermal treatment methods of producing few-layered graphene in terms of the production yield and/or the amount of graphite/graphene, using the data available in the literature, Table S3. Number of layers (NG) for FLG, Table S4. Sheet resistance of the PTFE and PET film measured by M3, Figure S1: TEM and HRTEM images of the BFPs, Figure S2. a: AFM images of the BFPs, Figure S3. SEM images of the powders; Figure S4. a: TEM images of the OBPs, b: TEM images of the BHPs, c: TEM images of the BFPs, d: TEM images of the FLG nanosheets, Figure S5. AFM image of the obtained products, Figure S6. Flake thickness distribution measured using AFM analysis of the FLG nanosheets., Figure S7. Image of the FLG nanosheets in pure water with the concentration of 20 mg/mL to 0.04 mg/mL., Figure S8. SEM images of the powders; a-b: A4 paper, e-f: PET, c-d: PTFE with the BFPs, g-h: PTFE with the FLG nanosheets.

## Abbreviations

FLG	few-layer graphene;
OBPs	the products were only obtained by ball milling without Fe_3_O_4_ nanoparticles;
BHPs	the products were obtained by the two-step method without Fe_3_O_4_ nanoparticles;
OHPs	the products were only obtained by hydrothermal treatment;
BFPs	the products were only obtained by ball milling with Fe_3_O_4_ nanoparticles;
BMPs	the products were obtained by ball milling with Fe_3_O_4_ nanoparticles in the two-step method;
PTFE	polytetrafluoroethylene;
PET	polyethylene glycol terephthalate.
