# Supplementary material for: High-Yield Production of Few-Layer Graphene via New-fashioned Strategy Combining Resonance Ball Milling and Hydrothermal Exfoliation
Source: Nanomaterials (Basel). 2020 Apr 2;10(4):667. doi: 10.3390/nano10040667 (PMC7221951; doi:10.3390/nano10040667)
Supplement: Supplementary file 1 [file nanomaterials-10-00667-s001.pdf]

nanomaterials MDPI

Supporting Information

High-yield production of few-layer graphene via newfashioned strategy combining resonance ball-milling and hydrothermal exfoliation

Qingfeng Yang[1], Ming Zhou*[123], Mingyang Yang[1], Zhixun Zhang[1], Jianwen Yu[1], Yibo Zhang[1], Wenjun Chen[1], Xuyin Li[1]

1State Key Laboratory of Tribology, School of Mechanical Engineering, Tsinghua University, Beijing 100084

2Key Laboratory for Advanced Materials Processing Technology, Ministry of Education, P.R.China

3Department of Industrial Engineering, Purdue University, 225 South University Street, West Lafayette, Indiana 47907, United States

* Correspondence: E-mail: zhouming@tsinghua.edu.cn.

1. Experimental conditions for preparation graphene using the two-steps method

Table S1. Experimental conditions for preparation of graphene

Materials	Step1: Ball milling	Step 2: Hydrothermal treatment	Results	Products label
Expanded graphite 20g	No nanoparticle Time :6h	--	Easy to coagulate in NMP (Figure **6**) AFM:32.69~263.00nm(Figure S5a).	OBPs
Expanded graphite 20g	No nanoparticle Time :6h	180℃ 3 h with 30 mL HNO_3	Easier to coagulate in NMP (Figure 6) AFM:15.74~82.60 nm(Figure S5b).	BHPs
Expanded graphite 0.5g	No	180℃ 3 h with 30 mL HNO_3	Bulk graphite. No effective exfoliated .	OHPs
Expanded graphite 20g	2.5g Fe_3O_4 nanoparticles Time : 6h	--	Heavy sediment after 7th days. (Figure 6). Few-layer graphene (Figure S1). AFM:~5.02 nm (Figure S5c)(after standing on the magnet holder and being centrifuged at 3000rpm)	BFPs
Expanded graphite 20g	2.5g Fe3O4 nanoparticles Time : 6h	180℃ 3 h with 30 mL HNO_3	highly stable for over 30th days. AFM: 100% less than 6 nm and 92% less than 3.5 nm (without centrifuged).	FLG

2. A comparison between different methods of producing few-layered graphene.

Table S2. A comparison between different ball-milling and hydrothermal treatment methods of producing few-layered graphene in terms of the production yield and/or the amount of graphite/graphene, using the data available in the literature

Amount of raw materials	Methods	Times	Product and Yield	Advantages / Disadvantages	Ref
300mg	Wet ball-milling	16h	0.29mg/ml graphene nanosheets /--	Low-cost, eco-friendly/ long time, less raw materials, low efficiency, no height information and exfoliation efficiency	1
0.6g	Ball-milling	20h	Few-layer graphene/ about 95% <5 layers(thickness distribution obtained by HRTEM counting the layers at the edges of 80 platelets.)	High efficiency/ long time, less raw materials, argon shield, only counted by HRTEM	2
1.49g /1.40g	Ball-milling	24h	GO >650 m2 g–1/ the carbon-based yields ranged from 86 to 97%	No harsh chemicals, low cost/ long time, no height information and exfoliation efficiency	3
Mass ration=1：4（no mass information）	Plasma-assisted ball milling	8h	Few-layer graphene< 10 layers/--	Eco-friendly/high voltage, no exfoliation efficiency, no flakes thickness distribution	4
75mg	Ball-milling	2.5h	Few-layer graphene thickness less than 10 nm, corresponding to 7-24 layers/ Production yield ~100%	Less time/ DY50 easily induced to explode, less raw materials, no flakes thickness distribution	5
5g	Ball-milling	45h	Multi-layer graphene(<10 layers)/--	Eco-friendly/ long time, argon shield, no exfoliation efficiency, no flakes thickness distribution	6
0.2g	Ball-milling	30h	Few-layer graphene 1.5 ~3 nm/--	Eco-friendly/ long time, no exfoliation efficiency, no flakes thickness distribution	7
2.0g	Ball-milling	24h	Few-layer graphene ~1.0nm/--	Eco-friendly/ long time, no exfoliation efficiency, no flakes thickness distribution, complex operation (10 min pause every 20 min)	8
10 mg mL−1	Wet ball-milling	6h	The estimated average number of layers per graphene= 4.4/ The corresponding graphene yield is about 26%.	Less time/low exfoliation efficiency, no flakes thickness distribution	9
0.8g	Wet ball-milling	10h	Few-layer graphene ≤ 10 layer/ 0.0085mg/ml·h	Low-cost, less time/ less raw materials, no exfoliation efficiency, no flakes thickness distribution	10

Amount of raw materials	Methods	Times	Product and Yield	Advantages / Disadvantages	Ref
8g	Ball-milling	24h	Few-layer graphene/--	Eco-friendly/ long time, no exfoliation efficiency, no flakes thickness distribution	11
some（no mass information）	Ball-milling	24h	Few-layer graphene 2~10layers/--	Low-cost, eco-friendly/ long time, no exfoliation efficiency, no flakes thickness distribution, complex operation (every 2 h opening the jar)	12
25g	Ball-milling	24h	Graphene nanosheets: HRTEM < 5layers, AFM< 2nm/--	Low-cost, eco-friendly/ long time, no exfoliation efficiency, no flakes thickness distribution	13
5g	Ball-milling	48h	Multi-layers graphene/--	Low-cost, eco-friendly/ long time, no exfoliation efficiency, no flakes thickness distribution	14
1g	Ball-milling	48h	Few graphene/--	Low-cost, eco-friendly/ long time, no exfoliation efficiency, no flakes thickness distribution	15
0.01 g mL/L	Ball milling,	30h	Few-layer graphene sheets＜5 layers /--	Eco-friendly/ long time, no exfoliation efficiency, no flakes thickness distribution	16
1.9g	Hydrothermal reaction+ stirred	4-10h	single- or few-layer graphene/ quantitative yields ~10 wt%	Low-cost, eco-friendly/ low yield, no exfoliation efficiency, no flakes thickness distribution	17
0.25g	Hydrothermal reaction	24h	single- or few-layer graphene/ yields ~8.2 wt%	Low-cost, environmentally friendly/ long time, low yield	18
0.08 mol/L	Hydrothermal reaction	12h	Few-layer graphene(8–12 single-layer graphitic nanosheet)/--	Easier economical/ long time, no exfoliation efficiency, no flakes thickness distribution	19
0.5g	Hydrothermal reaction	10h	The obtained ultrathin graphite nanostructure :2-10 layers, AFM : about 5 nm/--	Easier economical/ complex for sample pre-preparation, no exfoliation efficiency, no flakes thickness distribution	20
20g	Ball-milling 6h + Hydrothermal reaction 3h	9h	Few-layer graphene/ AFM: 92% ≤3.5 nm. HRTEM: 92% ≤10 layers.	High exfoliation efficiency (up to 92% ≤10 layers), high output rate (up to 85.26%), less ball milling time (6h) and amounts of raw materials	**This work**

3. Characterizations of the obtained products at different experiment conditions

The obtained powders by resonant ball milling with Fe_3O_4 nanoparticles(BFPs) were dispersed in NMP with the concentration of 0.1mg/ml. After standing on the magnet holder for 24 hours, the solution was centrifuged at 3000 rpm, and the supernatant was collected for TEM and AFM characterization. As shown in Figure S1, S2 and Figure S5c, we found that the edge sizes were 2-10 layers and the height of sample was less than 5.02 nm.

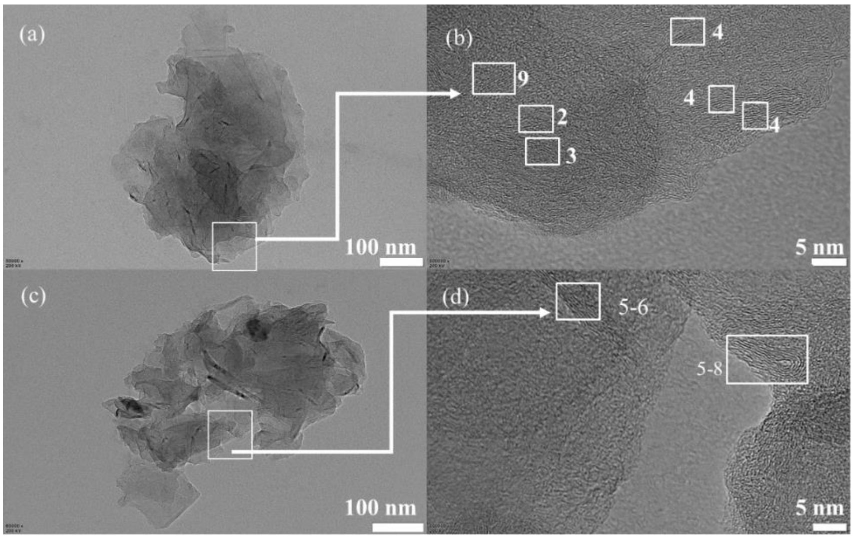

Figure S1. TEM and HRTEM images of the BFPs.

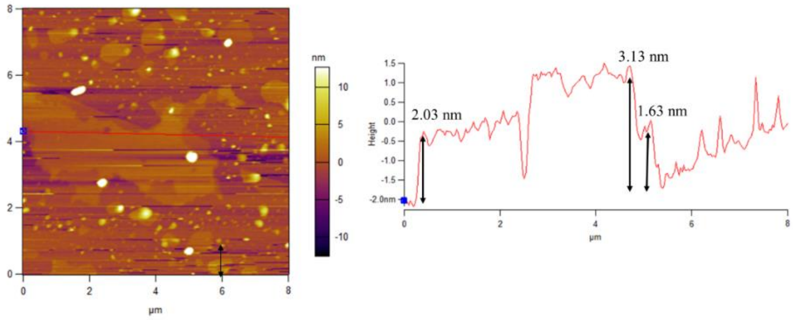

Figure S2. a:AFM images of the BFPs

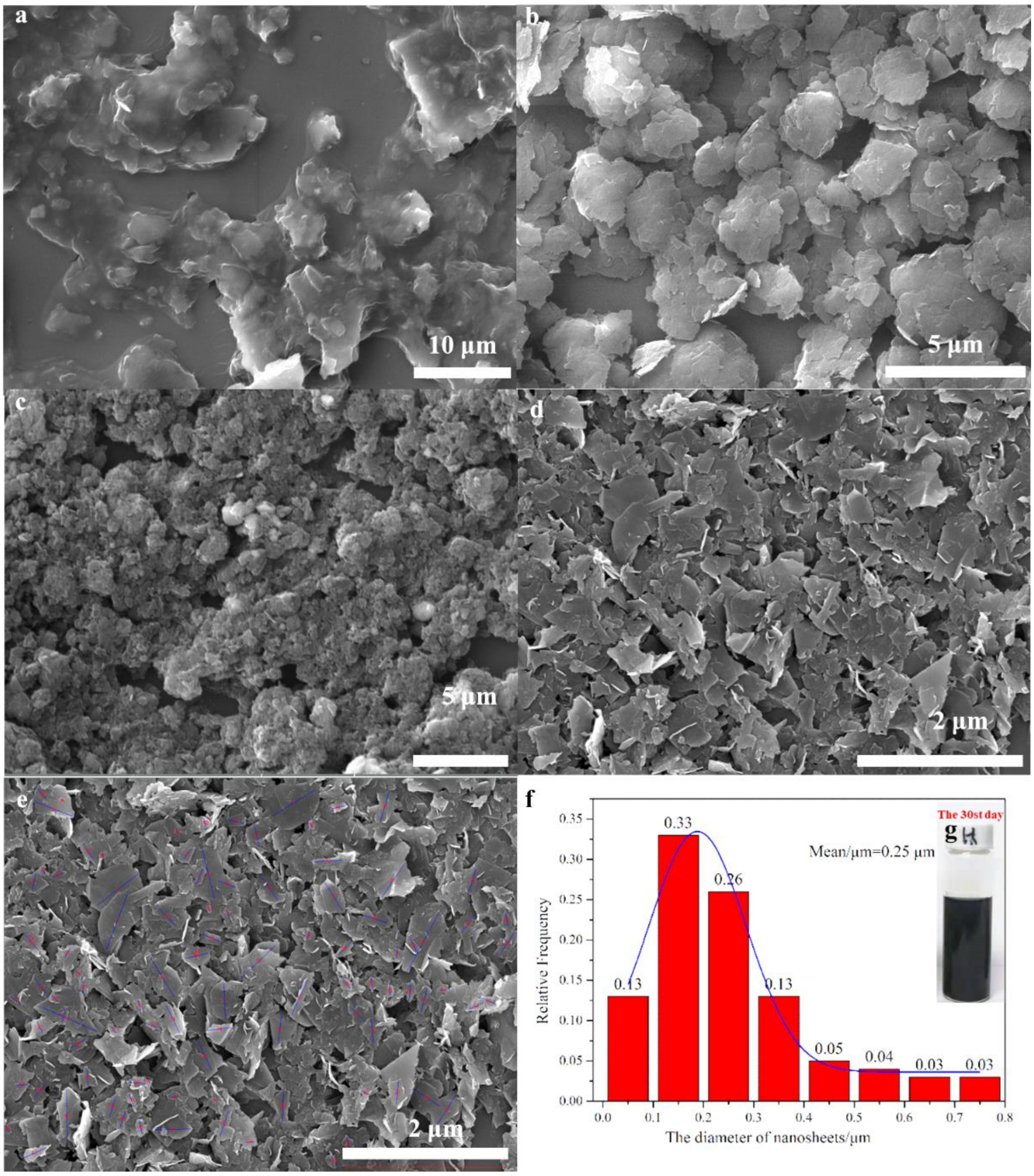

Figure S3. SEM images of the powders; a: the OBPs, b: the BHPs, c: the BFPs, d: the FLG nanosheets, e: the analysis of the image d by the nanomeasure software, f: the lateral dimension distribution of image e measured by nanomeasure software, g: image of the FLG nanosheets in NMP with the concentration of 0.1 mg/ml after 30th days.

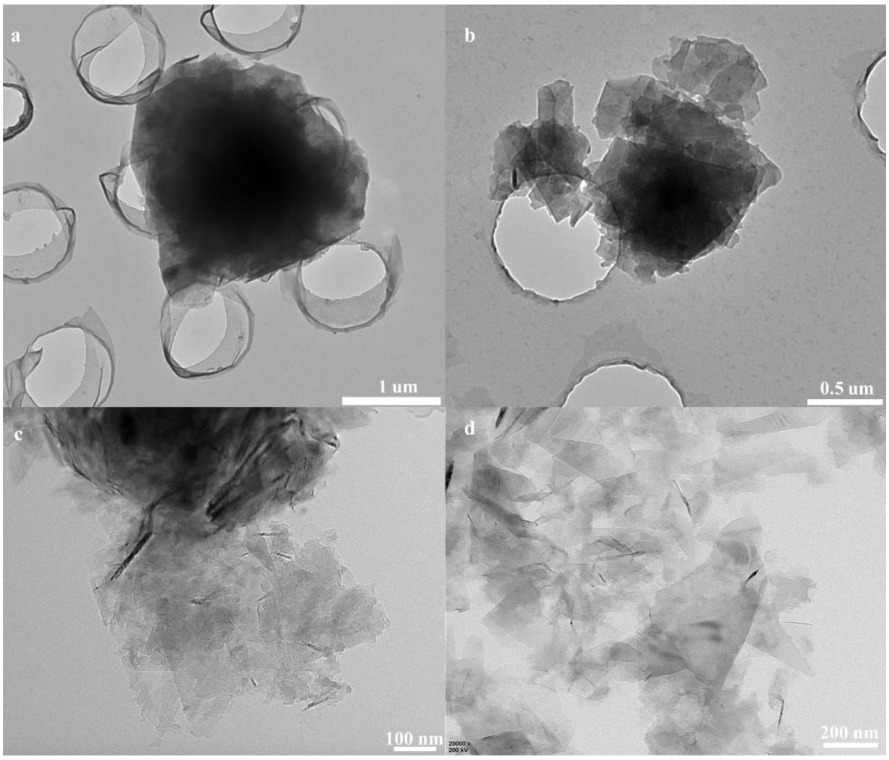

Figure S4. a: TEM images of the OBPs, b: TEM images of the BHPs, c: TEM images of the BFPs, d: TEM images of the FLG nanosheets.

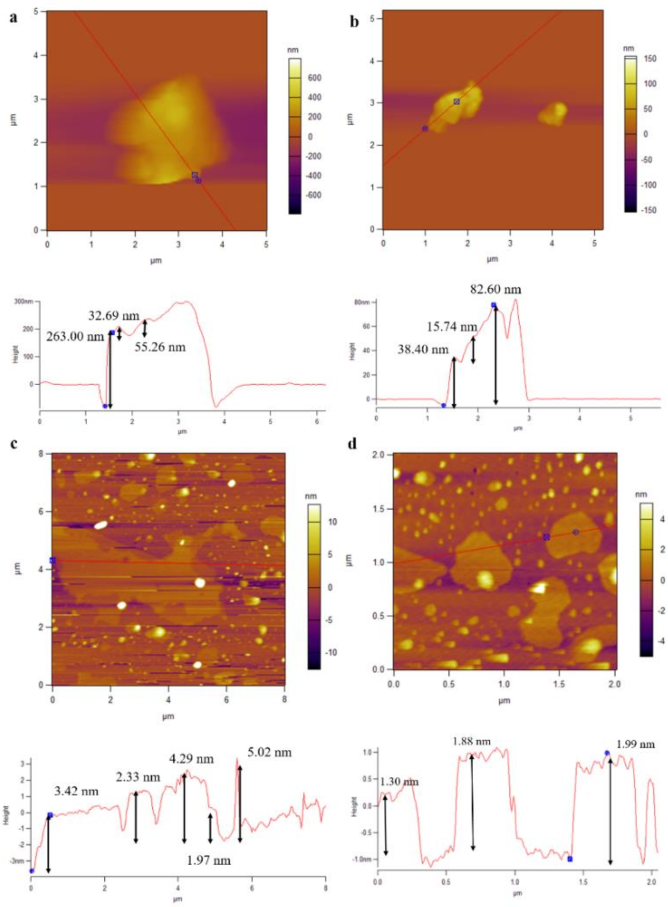

Figure S5. AFM image of the obtained products; a: the OBPs, b: the BHPs, c: the BFPs, d: the FLG nanosheets.

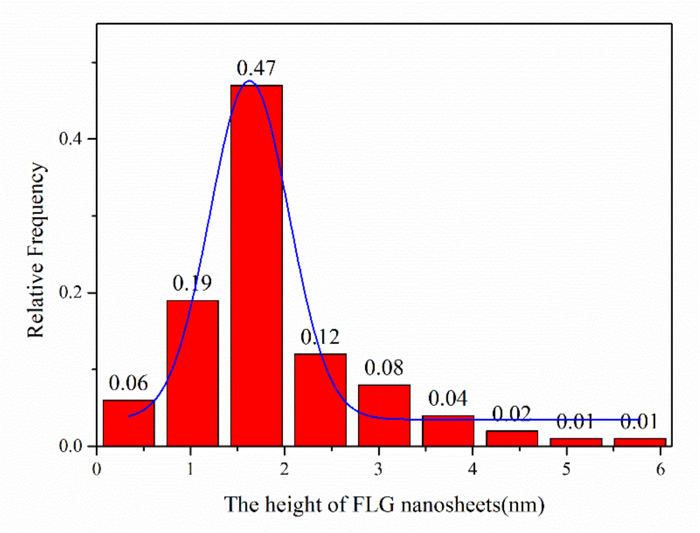

Figure S6. Flake thickness distribution measured using AFM analysis of the FLG nanosheets.

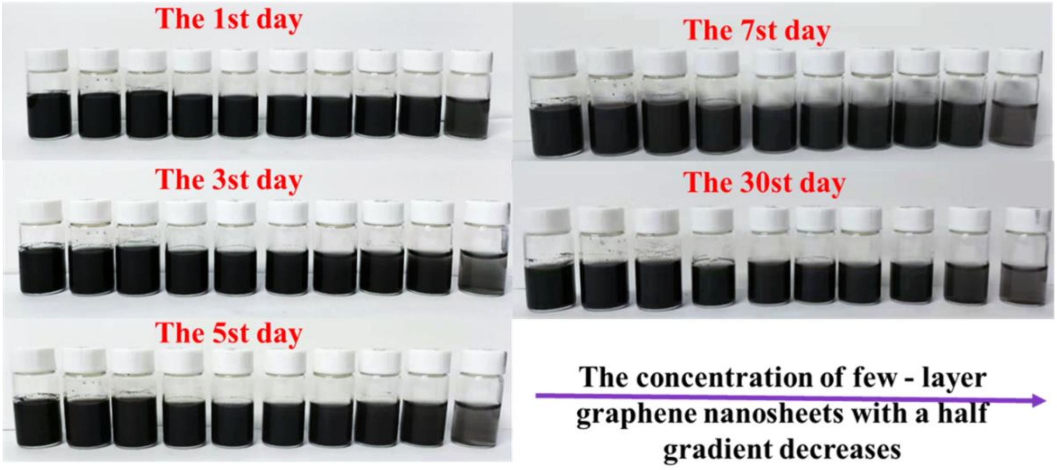

Figure S7. Image of the FLG nanosheets in pure water with the concentration of 20 mg/ml to 0.04 mg/ml.

4. Using Raman spectroscopy to measure flake thickness

The N_G (number of layers) of the FLG nanosheets were obtained by the additional Raman analysis. It is well known that the shapes of the 2D Raman bands (around 2700 cm^{-1}) reflect the thickness of the FLG. According to the formula obtained by Coleman and col.[9, 21], we calculated the N_G in our samples. The number layers of the prepared FLG nanosheets by two-steps method with Fe_3O_4 nanoparticles was calculated from comparison with the Raman spectrum of the initially expanded graphite material. We applied the following equation:

$$N_G = 10^{0.84M+0.45M^2} \quad (1)$$

Where M is equal to:

$$\mathrm{M} = \frac{I_{2Dene}(\omega=\omega_{p,2Dite})/I_{2Dene}(\omega=\omega_{s,2Dite})}{I_{2Dite}(\omega=\omega_{p,2Dite})/I_{2Dite}(\omega=\omega_{s,2Dite})} \quad (2)$$

Where I_{2Dene} and I_{2Dite} correspond with the intensity of 2D band for graphene and graphite, respectively. At least 20 individual Raman spectra of few-layer graphene nanosheets that were measured under the same test conditions were used to analyze

the calculations. The Raman results show that the FLG nanosheets had an average thickness of 7-8 layers.

Table S3. Number of layers (NG) for FLG

Sample	Band@2730.80cm^{-1} ($I_{2D,\omega p}$)	Band@2700.72cm^{-1} ($I_{2D,\omega s}$)	Relation $I_{2D,wp}/I_{2D,ws}$	M	N_G
Graphite	0.32	0.22	1.45	1	--
Graphene	0.32	0.29	1.09	0.76	7.94

5.Procedures for Depositing FLG nanosheets Coatings on Various Substrates

(1) *Deposition of FLG nanosheets films on PTFE membranes by filtration (see Figure 8a-b)*

0.3ml of 10 mg/mL auxiliary grinding (Fe_3O_4 nanoparticles) ball milling powders and FLG nanosheets aqueous solution was diluted with water to form 30 mL solution and filtrated on a PTFE membrane with diameter of 50 mm and pore size of 0.1 μm. The resulting FLG nanosheets coated PTFE film was dried at ambient conditions and its sheet resistance was measured with a four-point probe sheet resistance tester (M3, China).

(2) Deposition of FLG nanosheets conductive traces by hand drawing (see Figure 8c and 8e-g)

A liner dye brush was used for painting FLG nanosheets conductive traces on plastic pipe (Figure 8c), A4papers (Figure 8e), plant leaves (Figure 8f) and copper wire (Figure 8g) using the 10 mg/mL FLG nanosheets solution in ethanol as the ink. The wet FLG nanosheets traces were then dried at ambient conditions for further tests.

(3) Deposition of FLG nanosheets coatings on PET film by spin coating (see Figure. 8d)

150 μL of 10 mg/mL FLG nanosheets ink in ethanol was spread on a 3 cm × 3 cm PET film with 1000 ml pipette, and spin coated at 400 rpm for 30 s and then 2000 rpm for 30s. This spin coating process was repeated several times to obtain FLG nanosheets

coated PET film with average sheet resistance of about 51.80 ohm/sq, which was measured 5 times by M3 four-point probe sheet resistance tester.

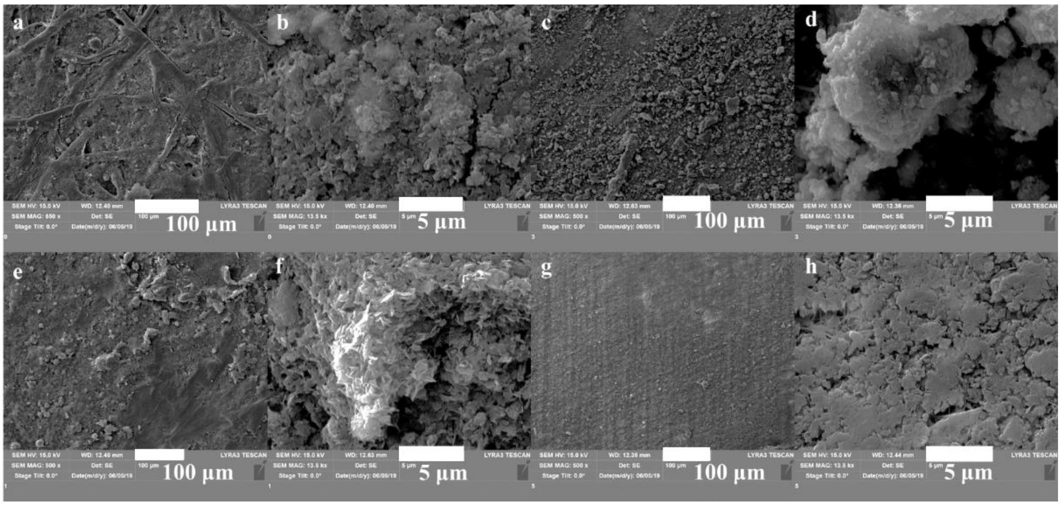

Figure S8. SEM images of the powders; a-b: A_4 paper, e-f: PET, c-d: PTFE with the BFPs, g-h: PTFE with the FLG nanosheets.

Table S4. Sheet resistance of the PTFE and PET film measured by M3.

Sample	Sheet resistance (Ω/sq)					Average sheet resistance (Ω/sq)
PTFE 1	4410.00	4580.00	4190.00	4210.00	4360.00	4350.00
PTFE 2	597.00	550.00	628.00	621.00	657.00	610.60
PET	57.30	47.50	48.50	52.20	53.50	51.80

Reference

1. Li, X.; Shen, J.; Wu, C.; Wu, K., Ball-Mill-Exfoliated Graphene: Tunable Electrochemistry and Phenol Sensing. *Small* **2019**, e1805567.
2. Cheng, C.; Jia, P.; Xiao, L.; Geng, J., Tandem Chemical Modification/Mechanical Exfoliation of Graphite: Scalable Synthesis of High-Quality, Surface-Functionalized Graphene. *Carbon* **2019**, *145*, 668-676.
3. Mahmoud, A. E. D.; Stolle, A.; Stelter, M., Sustainable Synthesis of High-Surface-Area Graphite Oxide Via Dry Ball Milling. *ACS Sustainable Chemistry & Engineering* **2018**, *6*, 6358-6369.
4. Lin, C.; Yang, L.; Ouyang, L.; Liu, J.; Wang, H.; Zhu, M., A New Method for Few-Layer Graphene Preparation Via Plasma-Assisted Ball Milling. *Journal of Alloys and Compounds* **2017**, *728*, 578-584.
5. Zhang, Z.; Sun, J.; Lai, C.; Wang, Q.; Hu, C., High-Yield Ball-Milling Synthesis of Extremely Concentrated and Highly Conductive Graphene Nanoplatelet Inks for Rapid Surface Coating of Diverse Substrates. *Carbon* **2017**, *120*, 411-418.

6. Liu, C.; Liu, X.; Tan, J.; Wang, Q.; Wen, H.; Zhang, C., Nitrogen-Doped Graphene by All-Solid-State Ball-Milling Graphite with Urea as a High-Power Lithium Ion Battery Anode. *Journal of Power Sources* **2017**, *342*, 157-164.
7. Balasubramanyan, S.; Sasidharan, S.; Poovathinthodiyil, R.; Ramakrishnan, R. M.; Narayanan, B. N., Sucrose-Mediated Mechanical Exfoliation of Graphite: A Green Method for the Large Scale Production of Graphene and Its Application in Catalytic Reduction of 4-Nitrophenol. *New Journal of Chemistry* **2017**, *41*, 11969-11978.
8. Sun, P.; Kuga, S.; Min, W.; Yong, H., Exfoliation of Graphite by Dry Ball Milling with Cellulose. *Cellulose* **2014**, *21*, 2469-2478.
9. Chao, T.; Dan, X.; Wang, J.; Zhi, Y.; Ren, G.; Ying, Z., Ultrahigh Conductive Graphene Paper Based on Ball-Milling Exfoliated Graphene. *Advanced Functional Materials* **2017**, *27*, 1700240.
10. Deng, S.; Qi, X.-d.; Zhu, Y.-l.; Zhou, H.-j.; Chen, F.; Fu, Q., A Facile Way to Large-Scale Production of Few-Layered Graphene Via Planetary Ball Mill. *Chinese Journal of Polymer Science* **2016**, *34*, 1270-1280.
11. Chua, C. K.; Sofer, Z.; Khezri, B.; Webster, R. D.; Pumera, M., Ball-Milled Sulfur-Doped Graphene Materials Contain Metallic Impurities Originating from Ball-Milling Apparatus: Their Influence on the Catalytic Properties. *Physical Chemistry Chemical Physics Pccp* **2016**, *18*, 17875.
12. Lv, Y.; Yu, L.; Jiang, C.; Chen, S.; Nie, Z., Synthesis of Graphene Nanosheet Powder with Layer Number Control Via a Soluble Salt-Assisted Route. *RSC Advances* **2014**, *4*.
13. Zhu, H.; Cao, Y.; Zhang, J.; Zhang, W.; Xu, Y.; Guo, J.; Yang, W.; Liu, J., One-Step Preparation of Graphene Nanosheets Via Ball Milling of Graphite and the Application in Lithium-Ion Batteries. *Journal of Materials Science* **2016**, *51*, 3675-3683.
14. Deb Nath, N. C.; Jeon, I.-Y.; Ju, M. J.; Ansari, S. A.; Baek, J.-B.; Lee, J.-J., Edge-Carboxylated Graphene Nanoplatelets as Efficient Electrode Materials for Electrochemical Supercapacitors. *Carbon* **2019**, *142*, 89-98.
15. Xue, Y.; Hao, C.; Jia, Q.; Dai, L., Nitrogen-Doped Graphene by Ball-Milling Graphite with Melamine for Energy Conversion and Storage. *2d Materials* **2015**, *2*, 044001.
16. Liu, Y.; Wu, H.; Chen, G., Enhanced Mechanical Properties of Nanocomposites at Low Graphene Content Based Onin Situball Milling. *Polymer Composites* **2016**, *37*, 1190-1197.
17. Liu, X.; Zheng, M.; Xiao, K.; Xiao, Y.; He, C.; Dong, H.; Lei, B.; Liu, Y., Simple, Green and High-Yield Production of Single- or Few-Layer Graphene by Hydrothermal Exfoliation of Graphite. *Nanoscale* **2014**, *6*, 4598-603.
18. Yang, S.; Zhuo, K.; Zhang, Z.; Liu, J.; Bai, G.; Wang, J., Sodium Fluoride-Assisted Hydrothermal Exfoliation of Graphite into Graphene as Filler of Epoxy Resin Coating to Protect Aluminum. *The Journal of Physical Chemistry C* **2019**, *123*, 27969-27977.
19. Wang, B.; Chang, Q. Y.; Gao, K., A Hydrothermal Reacting Approach to Prepare Few-Layer Graphene from Bulk Graphite. *Applied Surface Science* **2019**, *479*, 20-24.
20. Liu, P.; Si, Z.; Lv, W.; Wu, X.; Ran, R.; Weng, D.; Kang, F., Synthesizing Multilayer Graphene from Amorphous Activated Carbon Via Ammonia-Assisted Hydrothermal

Method. *Carbon* **2019**, *152*, 24-32.
21. Paton, K. R.; Eswaraiah, V.; Claudia, B.; Smith, R. J.; Umar, K.; Arlene, O. N.; Conor, B.; Mustafa, L.; Istrate, O. M.; Paul, K., Scalable Production of Large Quantities of Defect-Free Few-Layer Graphene by Shear Exfoliation in Liquids. *Nature Materials* **2014**, *13*, 624-630.
